# Impact of feeding and housing systems on disease incidence in dairy calves

**DOI:** 10.1136/vr.103895

**Published:** 2016-11-01

**Authors:** G. C. Curtis, C. McG. Argo, D. Jones, D. H. Grove-White

**Affiliations:** 1 Department of Obesity and Endocrinology, Institute of Ageing and Chronic Disease, University of Liverpool, Leahurst Campus, Neston, Wirral CH64 7TE, UK; 2 School of Veterinary Medicine, University of Surrey, Guilford GU2 7XH, UK; 3 Institute of Veterinary Science, University of Liverpool, Leahurst Campus, Neston, Wirral CH64 7TE, UK

**Keywords:** Calves, Nutrition, Husbandry, Diarrhoea, Pneumonia

## Abstract

Contentious issues in calf rearing include milk feeding level and single versus group housing. The current study was performed on a high-producing 170 Holstein cow dairy farm to investigate the impact of nutrition and housing on disease incidence. Calves (n=100) were allocated in birth order to one of two commonly used feeding strategies. Group A calves were group housed from birth and fed ad libitum milk replacer (MR) via a computerised machine using a single teat, with weaning commencing at 63 days. Group R calves were initially housed in individual pens receiving 2.5 litres of MR twice daily via a bucket until three weeks of age when they were group housed and fed 3 litres of MR twice daily via a group trough with weaning commencing at 56 days. In total, 80 (80 per cent) calves suffered from at least one incident of disease during the period from birth to 12 weeks. Group A calves had a greater risk of disease than group R calves (diarrhoea: OR 3.86 (95 per cent CI 1.67 to 8.9); pneumonia: OR 5.80 (95 per cent CI 2.33 to 14.44)). There was a 5.1 per cent incidence of failure of passive transfer of Ig assessed via measurement of plasma total protein concentrations at 48 hours of age. It is hypothesised that the increased diarrhoea risk in group A calves was most likely associated with group housing, while the increased pneumonia risk was associated with the use of a single teat allowing increased transmission of pathogens from calf to calf.

Increased milk feeding of dairy calves is associated with improved growth rates during the preweaning period and improved production and health in adult life (Soberon and others 2012, Soberon and Van Amburgh 2013). However, there is controversy regarding group housing of young dairy calves due to the greater potential for disease transmission compared with single-housing (Cobb and others 2014). Furthermore, individual penning of dairy calves and restricted milk or milk replacer (MR) feeding before weaning may present potential welfare problems (Stull and Reynolds, 2008). The chief diseases seen in preweaned dairy calves are diarrhoea during the first three weeks of life, and pneumonia, associated with viral and bacterial agents, generally occurring in calves over four weeks old (Lorenz and others 2011).

Various means of increasing milk or MR intakes in calves have been used, including increased concentrations and/or volume of MR fed twice daily, ad libitum acidified milk feeding, computerised feeding whether regulated or ad libitum (Appleby and others 2001, Drackley and others 2007, Borderas and others 2009, Anderson 2011, Hill and others 2013). The use of computerised feeding systems necessitates group housing of calves, a recognised risk factor for disease transmission (Svensson and Liberg 2006, Cobb and others 2014). A further issue is that on many farms assembling groups of 25–30 calves as recommended by manufacturers of these systems involves a wide range of age-mixing, thereby increasing the risk of pneumonia due to the likely range of immune competencies in large age groups.

The following study investigates the null hypothesis that disease risk among calves group housed from birth and fed via a computerised feeding system to receive ad libitum MR, was no different to that among calves initially individually housed than group housed, which received a fixed amount of MR twice daily, via either a single bucket or a group milk trough.

## Materials and methods

This study was performed in compliance with Home Office (Animal (Scientific Procedures) Act) legislation and was approved by the University of Liverpool Animal Welfare Committee. The study was performed between January 2011 and January 2013 at the University of Liverpool's Wood Park Dairy Farm, Neston, Wirral, UK (53°N). The farm milked approximately 170 Holstein Friesian cows, calving all year round, with an annual lactation yield of 10,500 litres on a three times daily milking regimen. All lactating cows were housed in freestalls all year round apart from cows in the final 100 days of lactation during the summer months that would graze outside. All non-lactating pregnant cows were housed throughout the eight-week dry period, initially in freestalls for five weeks then in a straw yard accommodating 5–15 cows. All calves were born in this straw yard. Calves born between 08:00 and 18:00  were removed from their mothers within four hours of birth and taken directly to the calf accommodation. Calves were all housed in the same pitched roof building, an adapted collecting yard and milking parlour. Calves born between 18:00 and 08:00 remained with their dam for up to 12 hours before transfer. All calves received between 3 and 4 litres of colostrum via an oesophageal feeder as soon as possible after birth. Calves all received further, freshly collected, dam-specific colostrum (fed via individual bucket) twice daily (2 litres per feed) for the first four days of life. MR (96.97 per cent dry matter (DM), 22.17 per cent crude protein, 19.76 per cent oil, 7.02 per cent ash, metabolisable energy (ME) 21.57 MJ/kg DM, pH 5.96, Blossom Easy Mix, Volac) was mixed and fed at 125 g/l at 37°C. Calves were assigned to one of two MR feeding strategies on arrival at the calf house in alternate groups of ≤6, such that each group of calves had an age range of no more than 14 days. Group A: ad libitum MR access (n=50); or group R: restricted MR access (n=50). For calves in group A, familiarisation and training for use of the automatic computerised teat feeder (Vario feeder, Forster Technik) from which ad libitum MR was dispensed began from birth. Calves in this group accessed MR from birth in addition to the four-day dam-specific colostrum meals. Group R calves received 2.5 litres MR twice daily for 21 days via individual buckets when it was increased to 3.0 litres fed twice daily via a group trough. Group R calves were exclusively fed dam-specific colostrum meals for the first four days of life, from July 2011 onwards.

**FIG 1:**
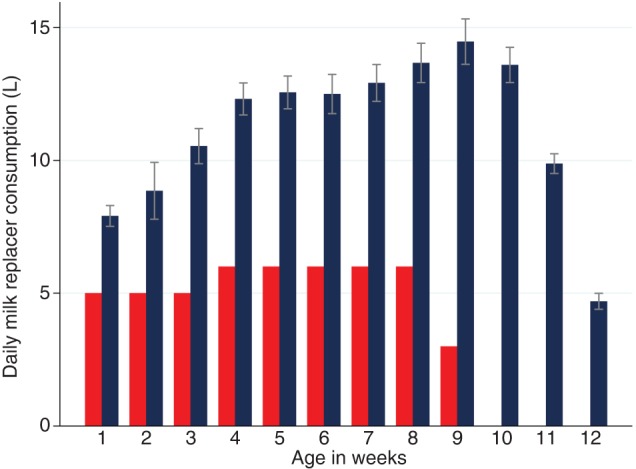
Mean (95% CI) daily milk replacer (MR) consumption for calves in group A (blue bar) and MR allowance for group R animals (red bar) from birth until 12 weeks of age

Group R calves were initially housed individually in metal-gated pens (1 m×2 m) over raised slatted flooring, bedded with wheat straw, from birth until 21 days of age, when they were moved to deep wheat straw bedded group pens (5 m×6 m) (n≤6, age range ≤14 days). Group A calves were grouped by age (n≤6, age range ≤14 days) and directly introduced to group pens on entry into the calf house. All calves had ad libitum access to forage (grass hay), fresh water and coarse mix concentrate feed up to a maximum of 2.5 kg per head daily. Group A calves were weaned by stepping down MR intake over a 21-day period commencing at 63 days, while group R calves were similarly weaned over a 7-day period commencing at 56 days of age. Mean MR intakes for both groups of calves are shown in Fig 1. All calves remained in the calf house until 14 weeks of age at which time the calves exited the study. All pens were bedded up weekly, and cleaned and disinfected with FAM disinfectant (FAM 30 Evans Vanodine International, UK) at a dilution rate of 1:200 between groups.

The specific gravity of the initial colostrum meal was assessed at collection with a Brix refractometer (Animal Reproduction Systems). Adequacy of passive transfer of Ig was assessed by measuring calf plasma total protein (PTP) concentrations at 48 hours of age using a refractometer taking a cut-off of 56 g/l as indicative of failure of passive transfer (FPT) of Ig (MacFarlane and others 2014).

All calves were examined twice daily for signs of illness by the researchers. Diarrhoea was diagnosed on the basis of a faecal score >2 (McGuirk 2008). Pneumonia was diagnosed on the basis of the presence of one or more of the following signs, accompanied by a rectal temperature >39.4°C: nasal discharge, ocular discharge, coughing and increased respiratory rate. Cases of diarrhoea received 2 litres twice daily of oral rehydration solution (Effydral, Zoetis) by bucket and teat or oesophageal feeder if no suck reflex was present. Rehydration therapy was continued for at least three days. Diarrhoeic calves were not removed from their accommodation and continued being fed MR at the same rate as their healthy counterparts. Cases of pneumonia were treated once with subcutaneous injections of tulathromycin (Draxxin, Zoetis) at a dose rate of 2.5 mg/kg and meloxicam (Metacam, Boehringer Ingelheim) at a dose rate of 0.5 mg/kg.

Data were collated in an Excel (Microsoft) spreadsheet and transferred to Stata V.13 (StataCorp) for analysis. Student's *t* and Fisher's exact chi-square tests were used to investigate any group differences in birth weight, colostrum quality, PTP concentrations and associations between colostrum quality, FPT and disease occurrence. Following exploratory univariable logistic regression analysis, two multivariable logistic regression models were fitted with the binary outcome variables being ‘occurrence of a case of diarrhoea (model 1) or pneumonia (model 2)’. To account for clustering of calves at group pen level, robust standard errors (se) and 95 per cent CIs were estimated. Only primary incident cases were considered in the analyses. All potential explanatory variables, namely feeding regimen (ad libitum v restricted fed), dam parity, colostrum quality, PTP, birth weight and group size, were offered to the initial models. Selection of variables for the final multivariable models was by backwards stepwise removal taking a P value <0.15 (log-likelihood test) for retention of a variable. Kaplan-Meier survival plots were fitted for age at pneumonia diagnosis. A log-rank test was used to compare survival plots. Where appropriate, standard deviations (sd) or 95 per cent CIs were estimated and presented. P values ≤0.05 were taken as indicating statistical significance.

## Results

Brix refractometry was performed on 90/100 peripartum colostrum samples. Mean (sd) specific gravity was 23.5 per cent (4.6). Using a cut-off of 22 per cent (Chigerwe and Hagey 2014) to indicate ‘good quality’ colostrum, 34 (37.8 per cent) samples failed to be classified as good quality. Colostrum quality was not associated with dam parity (P=0.32) or dietary group (P=0.52). PTP concentrations were measured in 97/100 calves at 48 hours of age. Mean PTP (sd) concentration was 68.9 g/l (0.81) with no dietary group (P=0.76) nor dam parity-associated (P=0.91) differences. Taking a PTP cut-off of 56.0 g/l as indicative of adequate passive transfer, five (5.1 per cent) calves were classified as having FPT. There were no associations between occurrence of FPT and calf group (P=1.00) or dam parity (P=0.65). There was a significant association between colostrum quality, based on a 22 per cent cut-off, and occurrence of FPT (P=0.045). There was no association between occurrence of FPT and occurrence of diarrhoea (P=0.39) or pneumonia (P=0.38). Median number of calves per group pen was 3 (range 2–6) for group R and 4 (range 2–6) for group A (P=0.13). There were a total of 34 groups of calves during the study.

Voluntary daily MR intakes in group A calves increased rapidly to reach 7.6 litres/day (95 per cent CI 6.5 to 8.7) by day 5. Thereafter, overall, mean daily MR intake increased linearly to reach 13.3 litres (95 per cent CI 12.4 to 14.2) by day 26 before the rate of increase slowed to peak at 15.3 litres/day (95 per cent CI 14.2 to 16.4) near the onset of gradual weaning on day 64. The maximum daily MR intake recorded for any calf was 25.5 litres. Over the three-week course of MR withdrawal, intakes declined at an overall average rate of 0.7 litres daily (Fig 1).

All calves presenting with clinical signs of disease were treated appropriately and the mortality rate was zero. In total, 80 (80 per cent) calves suffered from at least one incident of disease during the period from birth to 12 weeks. Calves in group R had an overall lower incidence of disease (n=33, 66 per cent, 95 per cent CI 52 to 80) than group A (n=47, 94 per cent, 95 per cent CI 87 to 100, P<0.001).

There were 57 cases of diarrhoea affecting 56 calves (one calf in group A had two separate incidents) equating to an overall primary case incidence of 56 per cent (95 per cent CI 46.1 to 66). There were significantly more primary cases of diarrhoea in group A (n=36, 72 per cent, 95 per cent CI 57.5 to 83.8) compared with group R (n=20, 40 per cent, 95 per cent CI 26.4 to 54.8, P=0.001). Rotavirus was isolated from 3/3 diarrhoeic faecal samples (two from group A and one from group R), suggesting that it was the most likely causal agent.

There were 42 cases of pneumonia affecting 37 calves (five calves (group A, four calves: group R, one calf)) suffered a second incidence of disease defined as occurring at least 14 days after the first treatment and requiring treatment, equating to an overall primary case incidence of 37 per cent (95 per cent CI 27.4 to 46.6). There were significantly more primary cases of pneumonia in group A (n=28, 56 per cent, 95 per cent CI 41.2 to 70) compared with group R (n=9, 18 per cent, 95 per cent CI 8.6 to 31.4, P<0.001).

Group A calves had a significantly higher risk of exhibiting symptoms of both diarrhoea and pneumonia, with unadjusted ORs of 3.86 (95 per cent CI 1.45 to 10.28) and 5.80 (95 per cent CI 1.35 to 24.86), respectively, compared with their group R counterparts. There was no association between dam parity, colostrum quality, PTP or birth weight and the likelihood of an episode of either diarrhoea or pneumonia, such that the only explanatory variable remaining in both models 1 and 2 was feeding group (ad libitum or restricted MR). For this reason, the results of univariable logistic regression analyses only are presented (Table 1).

**TABLE 1:** ORs (robust 95% CI) derived from univariable logistic regression models, including explanatory variables associated with the probability of a calf suffering an episode of either diarrhoea or pneumonia

Explanatory variable	OR	95% CI	P value
Outcome variable: episode of diarrhoea			
Feeding group (ad libitum v restricted milk replacer)	3.86	1.45–10.28	0.007
Dam parity (primiparous v multiparous)	1.41	0.64–3.12	0.40
Colostrum quality (%)	1.004	0.90–1.11	0.94
Plasma total protein (g/l)	1.35	0.83–2.20	0.26
Birth weight (kg)	0.98	0.91–1.05	0.55
Outcome variable: episode of pneumonia			
Feeding group (ad libitum v restricted milk replacer)	5.8	1.35–24.86	0.018
Dam parity (primiparous v multiparous)	1.40	0.61–3.24	0.13
Colostrum quality (%)	1.02	0.95–1.10	0.55
Plasma total protein (g/l)	1.01	0.68–1.50	0.94
Birth weight (kg)	0.98	0.91–1.06	0.62
Group size (per one calf increase in group size)	1.18	0.72–1.92	0.51

In the case of diarrhoea, all cases occurred in the first three weeks when group R calves were individually housed. Group A calves were all group housed from birth, with diarrhoea affecting calves in all groups (n=13), that is, all groups of group A calves experienced at least one case of diarrhoea. Mean within group diarrhoea prevalence among group A calves was 0.74 (95 per cent CI 0.58 to 0.89). Significantly more pens of group A calves were affected with pneumonia than was observed with group R calves (9/17 (69 per cent) v 5/17 (29 per cent), P=0.03). Mean within group pneumonia prevalence was significantly higher in group A calves compared with group R calves (0.58 (95 per cent CI 0.31 to 0.85) v 0.17 (95 per cent CI 0.014 to 0.33), P=0.006).

**FIG 2:**
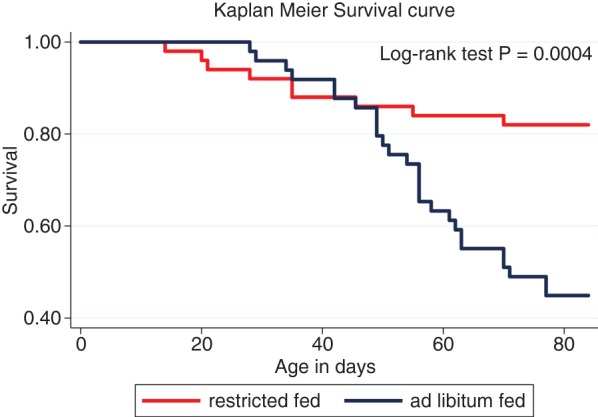
Kaplan-Meier survival plot of age in days of diagnosis of pneumonia

Mean age of onset of a case of diarrhoea in both feeding groups was 9.9 days (95 per cent CI 8.2 to 11.6 days, range 1–21 days). Mean age of diagnosis of pneumonia was significantly higher (P=0.003) in the ad libitum-fed calves compared with their restricted-fed counterparts (54 days 95 per cent CI 48.8 to 59.0 v 36 days 95 per cent CI 22.0 to 50.0) (Fig 2: log-rank test, P=0.0004). There was no association (P=0.81) between occurrence of a second case of pneumonia in an individual calf and feeding group. There was no association between occurrence of a case of diarrhoea and subsequent occurrence of a case of pneumonia in an individual calf (P=0.26).

**FIG 3:**
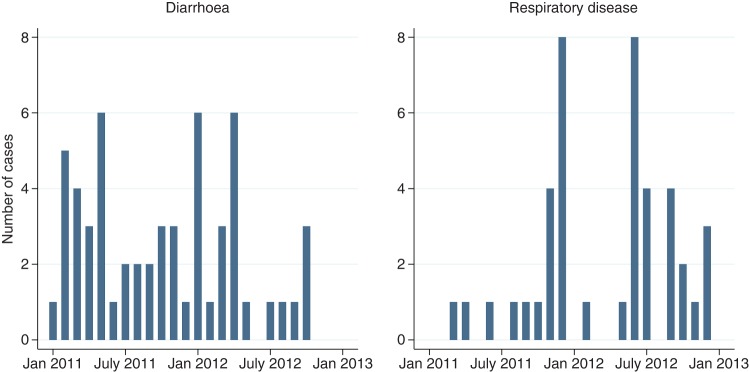
Total number of cases of diarrhoea and pneumonia throughout the study period (January 2011–January 2013)

**FIG 4:**
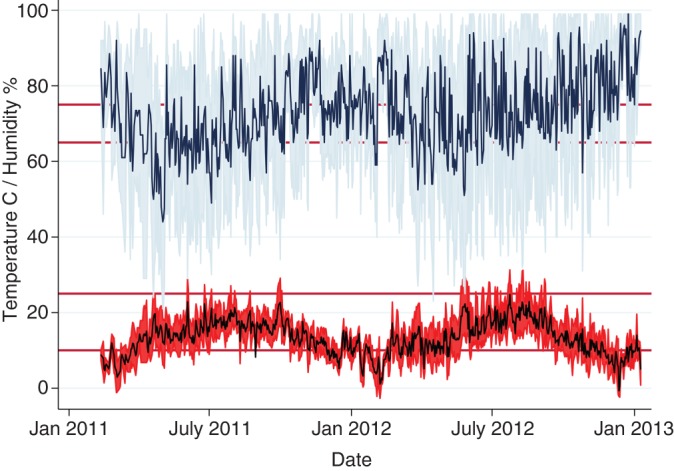
Mean temperature and humidity in the calf house for the duration of the study. Minimum and maximum temperature and humidity recordings are illustrated by the grey-shaded areas; reference lines at 10°C and 25°C and 65 per cent and 75 per cent depict optimum environmental temperature and humidity ranges, respectively, for young calves

There was considerable variation in the incidence of both diarrhoea and pneumonia throughout the study, although no clear trends were apparent. Diarrhoea was seen throughout most of the study period with only the final two months of the study being totally free from diarrhoea (Fig 3). The minimum and maximum temperatures and humidity's of the calf house were recorded daily (Fig 4) using a maximum and minimum temperature and humidity recording device placed in the middle of the house at a height of 1.5 m. Visual appraisal of Fig 3 suggests that the ambient temperature within the calf house was below the lower critical temperature (20°C: National Research Council (NRC) 2001) for a calf of up to three weeks of age for prolonged periods during the winter months. The relative humidity within the calf house fluctuated greatly and was above the optimum range for a young calf (65–75 per cent) throughout the study period with minor exceptions.

## Discussion

The results presented are nested within a larger study with the overall objective of comparing growth and performance between calves group housed with ad libitum feeding with calves kept under regimens currently commonly practised in the UK, namely individual housing for a period followed by group housing with restricted feeding of MR. Results from a concurrent questionnaire survey of 723 UK farmers (in preparation) suggested that such systems are commonplace although varying in precise timing, group size, etc.

The present study provides valuable information regarding impact of feeding and housing systems on disease incidence, despite there being implicit study limitations that must be acknowledged. First, calves were not randomly allocated to intervention arm. This was dictated by the farm size and calving rate, coupled with the requirement to produce groups of up to six calves with no more than 14 days age difference between them. Second, due to the standard farm policy for housing calves individually during the first three weeks of life, there is implicit confounding of feeding system by housing and vice versa, thus not allowing differences in disease risk to be attributed absolutely to either feeding system or housing system.

Diarrhoea and pneumonia are recognised as the key disease challenges facing the preweaned dairy calf and have major economic impacts, both in terms of mortality and reduced performance in later life (Roy and Ternouth 1972). While FPT of Ig is recognised as a key determinant (Furman-Fratczak and others 2011) of disease risk, there are other multiple factors affecting disease risk such as management strategies, nutrition, hygiene, temperature and humidity (Roy and Ternouth 1972, Haines and Godden 2011, Lorenz and others 2011). In the present study, disease incidence was high with 80 per cent of animals undergoing at least one disease episode, despite only 5 per cent of calves having evidence of FPT, re-enforcing the concept of disease being multifactorial involving infectious agents, host factors and environmental factors. The incidence rates for diarrhoea and pneumonia were high at 56 per cent and 37 per cent, respectively, with higher rates of both in the ad libitum-fed calves.

The calf house used in the present study was adapted for occupation by calves and suffered from a number of design faults including poor ventilation and drainage. These deficits were responsible for beds that were often wet despite regular addition of straw. Throughout the study period, the relative humidity in the calf house fluctuated, being outside the recommended range of 65–75 per cent for much of the time. During the winter months, the house temperature was often below 20°C (NRC 2001), the lower critical temperature for calves under three weeks of age. Thus, this suboptimal calf housing represented a significant basal risk factor for disease irrespective of feeding system employed. This is apparent from the high disease rates experienced by the group R calves (diarrhoea: 40 per cent; pneumonia: 18 per cent).

Diarrhoea was observed chiefly in the first three weeks of life and was likely associated with rotavirus infection, as determined by faecal sampling of three calves. Since no other calves were sampled, the role of other pathogens cannot be discounted. The diarrhoea incidence risk was considerably greater in the group housed, ad libitum-fed calves compared with the individually penned (for the first three weeks of life) restricted milk-fed calves (OR 3.86). Two hypotheses may be generated to explain this observation; first, feeding increased volumes of MR is a risk factor for diarrhoea. However, peer reviewed studies (Appleby and others 2001, Jasper and Weary 2002) do not support this hypothesis. The alternative hypothesis, supported by numerous studies, for example, Klein-Jobstl and others (2014), is that group housing of the ad libitum-fed calves facilitated transmission of infectious agents via direct contact and increased environmental contamination.

Thirty-seven calves suffered from one incident of pneumonia with five calves suffering from a second incident. Mean age of first occurrence was significantly higher in the ad libitum-fed calves compared with their restricted-fed counterparts (52.1 v 36 days). Diagnosis was made on clinical grounds and no laboratory investigations were performed. The high treatment success rate (100 per cent) was likely due to prompt recognition and treatment. From three weeks of age, all calves were housed under identical conditions in groups containing up to six calves with no more than 14 days age difference. These limits on group size and age range were in place specifically to minimise disease risk. The overall high pneumonia incidence (37 per cent) is likely a reflection of the suboptimal environmental conditions coupled with group housing of calves (Cobb and others 2014). The pneumonia risk was further increased in the ad libitum-fed calves both at pen level (OR 5.40) and at individual calf level (OR 5.80), suggesting that the practice of group feeding via a single shared teat compared with a trough represents an additional risk. In addition, bed wetness was accentuated by the increased amounts of urine produced consequent on the greater fluid intakes of the ad libitum-fed calves. The association between use of computerised feeding systems and increased incidence of pneumonia has been reported (Hepola 2003), although since use of such systems necessitates group housing, a known risk factor for pneumonia, it is unclear whether the increased risk is associated with use of a shared teat per se or associated with group housing. The present study would suggest that use of a shared teat is a risk factor in its own right, increasing the risk of pneumonia over and above the risk associated with group housing. It may be hypothesised that this is associated with transfer of respiratory pathogens in saliva and nasal secretions from calf to calf via the teat. This is supported by studies demonstrating oral infection with *Mycoplasma* species (Maunsell and others 2012) and by a report of isolating pathogenic *Mycoplasma* species from a shared teat on a farm with high levels of *Mycoplasma* species-associated pneumonia (J. Oultram, personal communication).

Despite the pneumonia risk being lower in the trough-fed calves compared with the teat-fed calves, they succumbed at an earlier age (36 v 52 days) than the ad libitum-fed calves. It is recognised that the decline in maternally derived antibodies is associated with increasing disease risk with the rate of decline depending, in part, on the amount of Ig consumed initially, as reflected by PTP concentrations measured in the first few days of life. In the current study, there was no difference in mean calf group PTP concentrations suggesting no association with the observed age-related disease susceptibility. There is increasing evidence of positive associations between nutrition status and immune response (Pollock and others 1994, Obeidat and others 2013) with improved nutritional status reducing the impact of clinical disease (Ollivett and others 2012). It is hypothesised that the increased nutrition provided to the ad libitum-fed calves resulted in increased resilience to respiratory pathogens such that clinical disease only occurred when calves were older despite the increased infectious risk associated with use of a single teat.

## Conclusions

Two main broad conclusions may be arrived at from the present study. First, group housing of calves during the first three weeks of life in suboptimal conditions may significantly increase diarrhoeal disease risk, likely due to increased transmission of enteric pathogens. Second, while automated feeder systems allow delivery of increased volumes of milk, facilitating improved growth, they appear to very significantly increase the risk of pneumonia if used in a suboptimal environment as in the present study. It would be prudent to avoid using automated feeding systems under such environmental conditions, that is, they should only be used in suitable housing systems that minimise the risk of pneumonia. In addition, there is an urgent need for manufacturers of such systems to develop teat sanitation protocols between individual calves to further minimise transmission.
